# CRTAM Protects Against Intestinal Dysbiosis During Pathogenic Parasitic Infection by Enabling Th17 Maturation

**DOI:** 10.3389/fimmu.2019.01423

**Published:** 2019-07-02

**Authors:** Luisa Cervantes-Barragan, Victor S. Cortez, Qiuling Wang, Keely G. McDonald, Jiani N. Chai, Blanda Di Luccia, Susan Gilfillan, Chyi-Song Hsieh, Rodney D. Newberry, L. David Sibley, Marco Colonna

**Affiliations:** ^1^Department of Pathology and Immunology, Washington University School of Medicine, St. Louis, MO, United States; ^2^Department of Molecular Microbiology, Washington University School of Medicine, St. Louis, MO, United States; ^3^Division of Gastroenterology, Department of Internal Medicine, Washington University School of Medicine, St. Louis, MO, United States; ^4^Division of Rheumatology, Department of Internal Medicine, Washington University School of Medicine, St. Louis, MO, United States

**Keywords:** mucosal immunity, T cells, interleukin 17, *Toxoplasma gondii*, CRTAM

## Abstract

The gastrointestinal tract hosts the largest collection of commensal microbes in the body. Infections at this site can cause significant perturbations in the microbiota, known as dysbiosis, that facilitate the expansion of pathobionts, and can elicit inappropriate immune responses that impair the intestinal barrier function. Dysbiosis typically occurs during intestinal infection with *Toxoplasma gondii*. Host resistance to *T. gondii* depends on a potent Th1 response. In addition, *a* Th17 response is also elicited. How Th17 cells contribute to the host response to *T. gondii* remains unclear. Here we show that class I-restricted T cell-associated molecule (CRTAM) expression on T cells is required for an optimal IL-17 production during *T. gondii* infection. Moreover, that the lack of IL-17, results in increased immunopathology caused by an impaired antimicrobial peptide production and bacterial translocation from the intestinal lumen to the mesenteric lymph nodes and spleen.

## Introduction

The gastrointestinal tract hosts the largest collection of commensal microbes in the body, which impact host metabolism as well as development and regulation of the immune system (1). Pathogenic infections at this site can cause significant perturbations in the microbiota, known as dysbiosis, that facilitate the expansion of pathobionts, elicit inappropriate metabolic and immune responses, and damage the barrier function of the intestine, ultimately causing pathology (2–5). This condition typically occurs during intestinal infection by *Toxoplasma gondii* (*T. gondii*), a widespread protozoan parasite of animals that also infects humans through the ingestion of oocysts contaminating water or food, or consumption of undercooked meat harboring tissue cysts (6). Host resistance to *T. gondii* depends on a potent IL-12-dependent IFN-γ response, which is largely mediated by Th1 cells (7–10). However, following oral infection with tissue cysts, the Th1-induced IFN-γ response to *T. gondii* destroys Paneth cells and blunts their capacity to produce antimicrobial peptides (AMPs), thereby impeding control of invasive *Enterobacteriaceae* (11–15).

In addition to triggering the IL-12-Th1-IFN-γ axis, oral infection by *T. gondii* also elicits a Th17 response. How Th17 cells contribute to the host response to *T. gondii* remains unclear. In two studies, abrogation of IL-17 signaling in *Il17ra*^−/−^ and *Il17a*^−/−^ mice resulted in increased susceptibility to *T. gondii* infection (16, 17). However, another study reported that *Il17ra*^−/−^ mice as well as B6 mice treated with a blocking anti-IL-17A antibody are more resistant to *T. gondii* infection (18). Finally, infection with a high dose of *T. gondii* cysts induced gut immunopathology independent of IL-17, but dependent on IL-22 (19).

We previously found that Th17 response to *T. gondii* depends on the cell surface molecule class I-restricted T cell-associated molecule (CRTAM) (20). CRTAM was originally described on activated CD8^+^ T cells and NK cells (21). It binds cell adhesion molecule 1 (CADM1), which is expressed on many myeloid and epithelial cells (22–27). We found that CRTAM is also expressed on intestinal intraepithelial CD4^+^ T cells upon activation. Moreover, we observed that *Crtam*^−/−^ and *Cadm1*^−/−^ mice had a selective defect in Th17 compared to wild-type (WT) mice during infection with the type II *T. gondii* strain Prugnaud (Pru), which is relatively non-pathogenic. However, they developed an effective Th1 response and cleared gut infection as effectively as WT mice (20). Thus, Th17 deficiency had no obvious consequences in this model of infection.

Here we challenged *Crtam*^−/−^ mice with an oral inoculation of tissue cysts of the type II ME49 strain of *T. gondii*, which is more pathogenic than the Pru strain we used previously, despite sharing the same genotype (28). Consistent with our previous study, *Crtam*^−/−^ mice controlled intestinal infection; they developed an effective Th1 response, but their Th17 response was impaired. Despite being able to control infection, *Crtam*^−/−^ mice suffered more pathology and many succumbed following infection. Remarkably, certain AMPs that are known to be induced by IL-17 including S100A8, S100A9, and beta defensins, were drastically reduced in the intestines of *Crtam*^−/−^ mice. As a result, mice lacking CRTAM were unable to control *T. gondii*-induced dysbiosis and subsequent bacterial translocation to the mesenteric lymph nodes and spleen. Paneth cell-derived AMPs, such as alpha defensins, were not affected, suggesting that control of *T. gondii*-induced dysbiosis requires a broad spectrum of AMPs. Although IL-17-producing CD4^+^ T cells were less abundant in *Crtam*^−/−^ mice than in WT mice, CD4^+^ T cells expressing RAR related orphan receptor t (Rorγt), the master transcription factor driving Th17, were equally represented; this suggests that CRTAM is required for terminal maturation of Th17 and acquisition of effector function rather than for Th17 lineage commitment. We conclude that CRTAM enables an optimal Th17 host response to pathogenic parasitic infections that is required for controlling dysbiosis and bacterial translocation associated with infection.

## Results

### *T. gondii* Infection Causes Marked Intestinal Pathology in *Crtam*^−/−^ Mice

**Figure 1:**
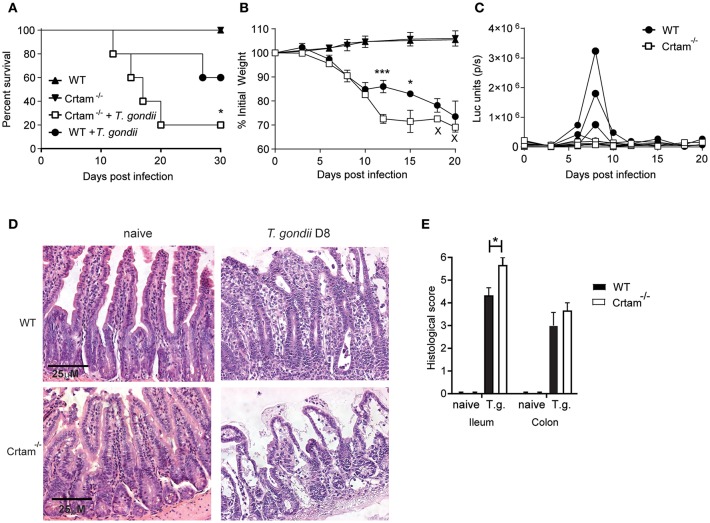
*Crtam*^−/−^ mice are more susceptible to pathology during *T. gondii* infection. WT and *Crtam*^−/−^ mice were infected orally with 10 cysts of *T. gondii* strain ME49, uninfected mice were used as control. **(A)** Survival **(B)** weight loss, and **(C)** parasite burden were analyzed at the indicated time points. **(D)** Representative sections of ileum stained with hematoxylin and eosin and **(E)** histological score in naïve mice and at day 8 post-infection. Symbols and bars in **(A,B,E)** represent means, error bars represent SEM. Symbols in **(C)** represent single mice. Data are representative of two independent experiments (*n* = 4–5). Statistical analysis was performed using Student's *t*-test or Log-rank Mantel-Cox test for survival curves (*p < 0.05; ***p < 0.001).

Given our previous observation that CRTAM has a limited impact on the response to a non-pathogenic strain of *T. gondii*, we wanted to re-examine CRTAM function in the context of intestinal infection by the more pathogenic ME49 strain. *Crtam*^−/−^ and WT mice were infected orally with 10 cysts of *T. gondii* ME49, which is capable of causing death at higher inoculum. This line of ME49 also expresses luciferase and therefore can be visualized by bioluminescence imaging of the whole mouse (29). *Crtam*^−/−^ mice lost more weight and more of them died following infection (Figures 1A,B), although not statistically significant, Crtam^−/−^ mice show a trend to control parasite replication better than WT (Figure 1C). Histological analysis of the intestine revealed that *Crtam*^−/−^ mice had more ileal pathology than did WT mice after infection with *T. gondii; Crtam*^−/−^ mice displayed loss of epithelial integrity, mucosal and submucosal edema, altered villus architecture, and moderate inflammatory infiltrates (Figures 1D,E), the colon was equally affected in WT and Crtam^−/−^ mice (Figure 1E; Figure S1). We conclude that the cause of increased lethality in *Crtam*^−/−^ mice is exacerbated pathology rather than uncontrolled infection.

### CRTAM Deficiency Impairs the Th17 Response to *T. gondii*

**Figure 2:**
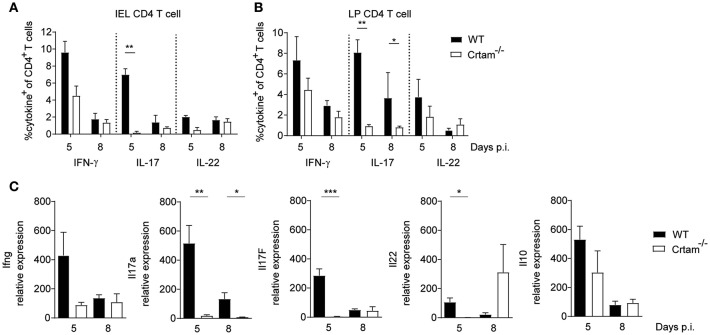
CRTAM deficiency results in an impaired IL-17 production by T cells. WT and *Crtam*^−/−^ mice were infected orally with 10 cysts of *T. gondii* strain ME49. Frequencies of **(A)** intraepithelial and **(B)** lamina propria IFN-γ, IL-17, and IL-22 producing CD4^+^ T cells were analyzed at days 5 and 8 post-infection. **(C)** Expression of *Ifng, Il17a, Il17f*, *Il22*, and *Il10* in the ileum of infected mice analyzed by quantitative PCR at days 5, and 8 post-infection. Bars represent means, error bars represent SEM. Data are representative of two independent experiments (*n* = 4). Statistical analysis was performed using Student's *t*-test (*p < 0.05; **p < 0.01; ***p < 0.001).

We next sought to investigate the mechanisms by which CRTAM counteracts intestinal pathology. No significant differences were observed in the frequencies of CD4^+^ T cells producing IFN-γ and IL-22 that were present within intraepithelial (IEL) and lamina propria (LPL) lymphocytes isolated from the small intestine of WT and *Crtam*^−/−^ mice after infection with *T. gondii*; however, considerably fewer CD4^+^ T cells producing IL-17 were detected in *Crtam*^−/−^ mice (Figures 2A–C). Moreover, cytokine profiles of the ilea of WT and *Crtam*^−/−^ mice after infection with *T. gondii* confirmed a strong reduction in the abundance of IL-17A and IL-17F mRNAs in *Crtam*^−/−^ mice, while the amounts of IFN-γ, IL-22, and IL-10 mRNAs were not significantly different between WT and *Crtam*^−/−^ strains. Together, these results suggest that CD4^+^ T cells require CRTAM in order to produce IL-17 during *T. gondii* infection.

### Lack of CRTAM Curtails the Production of AMPs That Restrain the Microbiota

**Figure 3:**
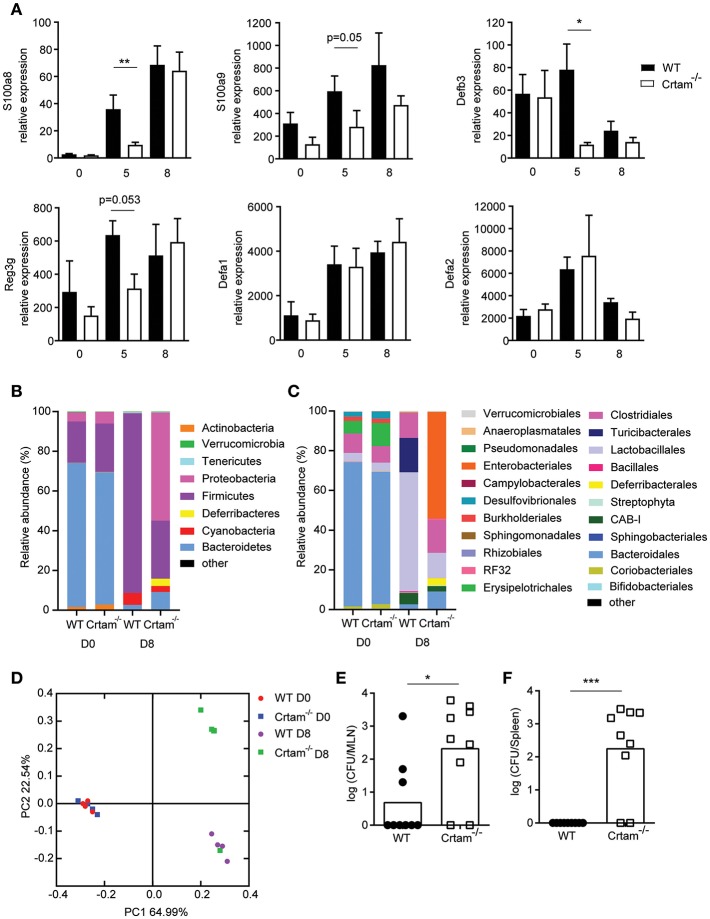
Absence of CRTAM expression results in impaired antimicrobial peptide production. WT and *Crtam*^−/−^ mice were infected orally with 10 cysts of *T. gondii* strain ME49. **(A)** Expression of *S100a8, S100a9, Defb3, Reg3g, Defa1*, and *Defa2* mRNA in the ilea of infected mice at days 0, 5, and 8 determined by quantitative PCR. 16S rRNA sequencing of ileal lumen contents at day 0 or 8 days post-infection (D8). Data are mean bacterial changes at the **(B)** phyla or **(C)** order level, and **(D)** principal coordinates analysis on weighted UniFrac distances (*n* = 4). Bacterial counts present in the **(E)** mesenteric lymph node and **(F)** spleen at day 8 post-infection. Bars represent means, error bars represent SEM. Data are representative of two independent experiments (*n* = 4–8). Statistical analysis was performed using Student's *t*-test (*p < 0.05; **p < 0.01; ***p < 0.001).

Given that the dysbiosis incurred during infection with *T. gondii* has previously been attributed to IFN-γ-induced death of Paneth cells and consequent loss of AMPs that results in an increase of Enterobacteriaceae (11), we assessed expression of various AMPs in the ilea of *Crtam*^−/−^ and WT mice infected with ME49. Significantly less *S100a8, S100a9, Regenerating islet-derived 3 gamma (Reg3g)* and *Defensin beta 3* (*Defb3*) mRNA was detected in *Crta*m^−/−^ mice than in WT mice at day 5 post-*T. gondii* infection. In contrast, *Defensin alpha 1* and *alpha 2* were equally expressed (Figure 3A). 16S rRNA sequencing analysis further showed that although naïve WT and *Crta*m^−/−^ mice had a similar microbiome (Figures 3B–D; Table S1), an increase in Enterobacteriales (Figure 3C; Table S1) in particular γ-proteobacteria (Figure S2) was observed in infected *Crtam*^−/−^ mice compared to WT (Figure 3B). Moreover, more bacteria were present in mesenteric lymph nodes and spleens from *Crtam*^−/−^ mice than in those from WT mice at day 8 post-infection (Figures 3E,F), suggesting that this partial defect in AMPs is sufficient to allow breach of the barrier and bacterial translocation, which may contribute to the death of *Crta*m^−/−^ mice. Remarkably, S100A8, S100A9, and Defb3 are primarily induced by IL-17, whereas defensin alpha 1 and alpha 2 are chiefly produced by Paneth cells. Our results suggest that the Th17 response to pathogenic challenge with *T. gondii* prevents dysbiosis by inducing AMPs for the containment of commensal bacteria.

### IL-17 Prevents Bacterial Translocation During *T. gondii* Infection

**Figure 4:**
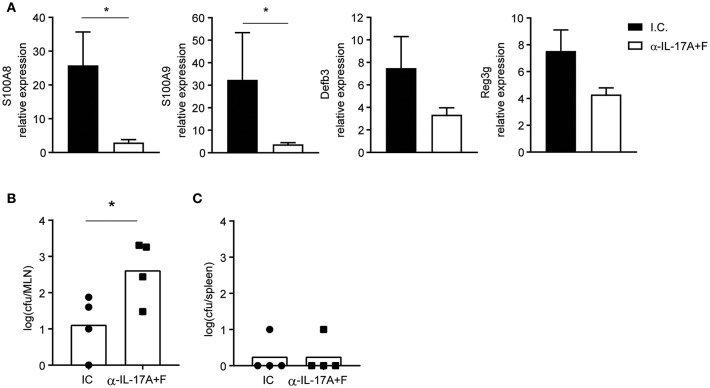
IL-17 blockade curbs AMP production and augments bacterial translocation. WT mice were injected intraperitoneally with 350 μg of anti-IL-17A and anti-IL-17F or an isotype control (I.C.) at days −1 and 5 and infected orally with 10 cysts of *T. gondii* strain ME49. **(A)** Expression of *S100a8, S100a9, Defb3*, and *Reg3g* mRNA in the ilea of infected mice at day 8 determined by quantitative PCR. Bacterial counts present in the **(B)** mesenteric lymph node and **(C)** spleen at day 8 post-infection. Bars represent means, error bars represent SEM. Data are representative of two independent experiments (*n* = 4). Statistical analysis was performed using Student's *t*-test (*p < 0.05).

To validate the importance of IL-17 for the production of AMPs and control of bacterial translocation during *T. gondii* infection, WT mice were treated with either anti-IL-17A and anti-IL-17F blocking antibodies or an isotype control and infected with 10 cysts of *T. gondii* ME49 (Figure 4). Mice that received the anti-IL-17 antibodies expressed less AMP mRNA and had more bacterial translocation to the mesenteric lymph nodes, confirming that IL-17 is essential for preventing dysbiosis. Of note, bacteria were not found in the spleens of mice treated with the anti-IL-17 blocking antibodies, suggesting that other cytokines or chemokines may contribute to barrier protection.

### CRTAM Engagement Is Required for Terminal Differentiation of Th17

**Figure 5:**
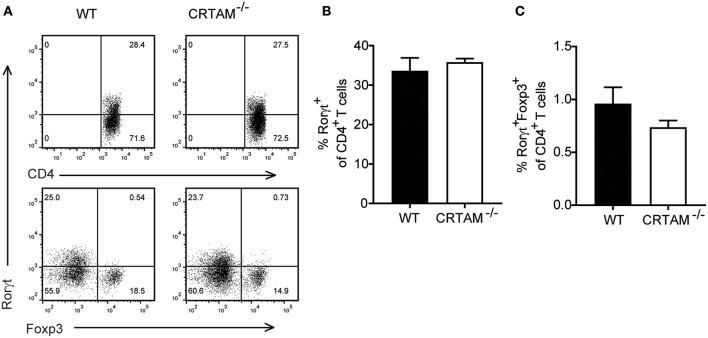
Th17 lineage specification is CRTAM-independent. WT and *Crtam*^−/−^ mice were infected orally with 10 cysts of *T. gondii* strain ME49. **(A)** Representative flow cytometry plots showing percent of rorγt of CD4 T cells and foxp3 and rorgt expression on CD4 T cells. **(B)** frequencies of Rorγt^+^ T cells and **(C)** Rorγt^+^Foxp3^+^ T cells in the small intestinal lamina propria of infected mice at day 5. Bars represent means, error bars represent SEM. Data are representative of two independent experiments (*n* = 3). Statistical analysis was performed using Student's *t*-test.

Th17 differentiation is a multi-step process that requires induction of the master Th17 transcription factor Rorγt in naïve CD4^+^ T cells (30, 31), followed by maturation of Rorγt^+^CD4^+^ T cells into IL-17 secreting cells, which depends on exposure to cytokines, such as IL-23 (32). To understand when CRTAM is required during Th17 development, we assessed expression of Rorγt in lamina propria CD4^+^ T cells during *T. gondii* infection in WT and *Crtam*^−/−^ mice. Rorγt^+^ T cells were present at similar frequencies in WT and *Crtam*^−/−^ mice (Figures 5A,B), indicating that CRTAM is dispensable for Th17 lineage specification.

Since the effector function of Rorγt^+^CD4^+^ T cells is controlled by a subset of regulatory T cells that express both Forkhead box p3 (Foxp3) and Rorγt and suppress intestinal inflammation (33, 34), we asked whether lack of CRTAM facilitates the development of Foxp3^+^Rorγt^+^ T cells. However, we didn't see an increase in Foxp3+Rorγt+T cells in *Crtam*^−/−^ mice (Figures 5A,C). These results suggest that CRTAM does not impact modulation of Th17 through Treg induction.

## Discussion

Host control of *T. gondii* infection has been shown to rely on a strong Th1 response (6, 7). Our study demonstrates that the Th17 response is important for host survival to intestinal infection by a more pathogenic type II strain of *T. gondii*. By secreting IL-17, Th17 support the production of AMPs, that prevents the dysbiosis and bacterial translocation associated with pathogenic infection. How Th17 response impacts oral infection by *T. gondii* has been controversial. While some studies have reported a protective role for IL-17 signaling during *T. gondii* infection (16, 17), other studies have found that IL-17 can either promote or is irrelevant for immunopathology (18, 19). We postulate that these discrepancies may be due to differences in the *T. gondii* strains used and doses administered in each study, or differences in the microbiota of the mice used. The ability of the various strains used to disrupt the microbiota and the susceptibility of the microbiota to perturbations induced by *T. gondii* clearly have a great impact; because the microbiota in genetically identical mice varies enormously in different mouse facilities, replicating any of these studies is challenging.

It has been reported that *T. gondii*-induced dysbiosis depends on the negative impact of IFN-γ produced by CD4^+^ T cells on Paneth cells and their ability to produce certain AMPs (11). Our study demonstrates that IL-17-induced AMPs contribute to provide a broad umbrella of protection from dysbiosis that is essential during pathogenic *T. gondii* infection; therefore a reduced Th17 response during infection results in exacerbated dysbiosis and pathology. Indeed, *Crtam*^−/−^ mice had a very selective reduction in AMPs induced by IL-17, but expressed AMPs released by Paneth cells at normal levels. Given the role of IL-17 in inducing the differentiation and recruitment of neutrophils (35), we envision that Th17 could potentially act by mobilizing a neutrophilic source of AMPs that, together with Paneth cells, provides the broad spectrum of AMPs required for preventing perturbation of microbiota, particularly the expansion of *Enterobacteriaceae*. The relative impacts of Paneth cell- and neutrophil-derived AMPs in preventing dysbiosis may vary depending on the type and location of the intestinal infection.

Our data support a role for CRTAM in intestinal Th17 responses, whereas Th1 and Th22 responses seem to be relatively CRTAM-independent. This conclusion is consistent with previous studies focusing on the impact of CRTAM on T cell responses in various contexts (24, 36–38), though it does not concur with the originally proposed role for CRTAM in CD4^+^ T cell production of IL-22 and IFN-γ (39). Our study further advances our understanding of the role of CRTAM in Th17; clearly it is not required for the initial induction of Rorγt in naïve CD4^+^ T cells, as *Crtam*^−/−^ and WT mice have similar percentages of intestinal Rorγt^+^CD4^+^ T cells. Rather, CRTAM seems to enable IL-17 production by Th17 cells. Since myeloid cells secrete IL-23 in response to changes in the intestinal microbiota (40, 41) and express the CRTAM ligand CADM1 (26), we speculate that CRTAM-CADM1 interactions may sustain the exposure of Rorγt^+^CD4^+^ T cells to myeloid cells producing IL-23, thereby promoting their final maturation into IL-17-producing CD4^+^ T cells.

In conclusion, our study demonstrates that a protective immune response against *T. gondii* must both effectively control invading pathogens through the Th1-IFN-γ axis and prevent dysbiosis, barrier leakage and microbial translocation through the Th17-AMP axis. Given that oral infection of mice with *T. gondii* causes intestinal inflammation and ileitis that resemble some aspects of Crohn's disease (42), our findings suggest that unbalanced representation of CD4^+^ T cell subsets may have a decisive impact in the onset and progression of human inflammatory bowel disease.

## Materials and Methods

### Mice

*Crta*m^−/−^ and C57BL/6 mice were bred in a pathogen-free facility at Washington University. Age and sex matched animals were used throughout the experiments and were co-housed from birth. All animal experiments were conducted according to U.S.A. Public Health Service Policy of Humane Care and Use of Laboratory Animals. All protocols were approved by the Institutional Animal Care and Use Committee (School of Medicine, Washington University in St. Louis).

### *T. gondii* Infection and Blocking Antibodies

The luciferase-expressing Me49-Luc type II *T. gondii* strain was provided by Laura Knoll (29). Tissue cysts were obtained from the brains of infected mice. Experimental mice were infected with 10 cysts of *T. gondii* Me49 orally. Weight was monitored every 48 h for the first 20 days. Parasite burden was analyzed by bioluminescence measurements. Mice were imaged every 48 h for 20 days by i.p. injection of 150 mg of luciferin D (Biosynth AG) per kg of body weight and using a Xenogen IVIS 100 (Caliper Life Sciences). Data was analyzed with the Living Image Software (Caliper Life Sciences) and is expressed in relative light units. Blocking antibodies anti IL-17A (IL-17F) and IgG1 isotype control were purchased from BioxCell. Mice were injected intraperitoneally with 350 μg at day −1 and +5 of *T. gondii* infection.

### Tissue Histology and Histological Score

Small intestines from mice were obtained and the luminal contents were flushed with cold PBS. The ileal portion was fixed in formalin, longitudinally embedded in paraffin. Five micrometer sections were cut and stained with hematoxylin eosin. Tissue slides were deidentified and scored as previously described (43). Briefly, sections were scored on three factors: (1) lymphocytes infiltration (0, no infiltration, 1, some infiltration, 2, massive infiltration in lamina propria, 3, massive infiltration in lamina propria and muscle) (2) edema (0, no edema, 1, edema <50 μm, 2, edema between 50 and 100 μM, 3, edema >100 μm), and (3) ulceration (0, no ulceration, 1, <10% epithelium ulcerated, 2, between 10 and 20% epithelium ulcerated, 3, >20% epithelium ulcerated). Histology was scored in a blinded fashion and scores were totaled for a cumulative score between 0 and 9.

### Tissue Isolation, Flow Cytometry, and Cell Sorting

Small intestine intraepithelial lymphocytes (siIEL) and lamina propria lymphocytes (siLPL) were prepared at day 5 and 8 post-infection as described by Lefrançois and Lycke (44). For flow cytometry, the following fluorophore-labeled monoclonal antibody (mAbs) were used: CD3 (145-2C11), CD8 (53–6.7) from BD Biosciences; CD4 (GK1.5) from eBioscience; and CD45 (30F11.1) from Biolegend. For intracellular stainings, cells were stimulated with the Cell Activation cocktail without brefeldin (Biolegend) for 4 h in the presence of Golgi Plug (BD Biosciences). Surface staining was performed, followed by fixation and permeabilization (BD Cytofix cytoperm Plus kit, BD biosciences). The monoclonal antibodies IL-17A (eBio17B7) from eBiosciences, IFN-γ (XMG1.2) and IL-22 from BD Biosciences were used. For rorgT staining, surface staining was performed, followed by fixation with the eBioscience foxp3 transcription factor buffer set (ebiosciences). The monoclonal antibody anti-Rorγt (AFKJS-9) and foxp3 (FJK-16s) from ebiosciences was used. Samples were processed in a FACSCantoII (BD Biosciences) and analyzed with FlowJo Software (Flowjo LLC). Flow cytometric cell sorting was performed using a FACSAria II (BD Biosciences).

### Bacterial Counts in Tissue

Mesenteric lymph nodes and spleens from infected mice were aseptically removed and homogenized in PBS in a Magnalyzer (Roche; 5,000 rpm × 30 s). The homogenized organs were plated in Luria Bertani (LB) agar and incubated at 37°C for 48 h. Colonies in the plates were counted and the colony forming units per organ calculated.

### Fecal DNA Extraction, Tissue RNA Extraction, and Quantitative PCR

Fecal DNA was extracted using a QIAmp DNA Stool mini kit and RNA was extracted from ileum samples with an RNeasy Mini kit following manufacturer's recommendations (Qiagen). cDNA was synthesized from RNA with Superscript III first-strand synthesis system for RT-PCR (Invitrogen). RNA expression was analyzed by quantitative PCR using Universal SYBR Green PCR Master Mix (Bio-Rad Laboratories) and an ABI7000 (Applied Biosystems). The following oligonucleotides were used: Il17a Fwd 5′-AGAGCTGCCCCTTCACTTTC-3′, Il17a Rev 5′-TGGGGGTTTCTTAGGGGTCA-3′, Il17f Fwd 5′-CTGGAGGATAACACTGTGAGAGT-3′, Il17f Rev 5′-TGCTGAATGGCGACGGAGTTC-3′, Il22 Fwd 5′-GCTCAGCTCCTGTCACATCA-3′, Il22 Rev 5′-AGCTTCTTCTCGCTCAGACG-3′, ifng Fwd 5′-ACAATGAACGCTACACACTGCAT-3′, ifng Rev 5′-TGGCAGTAACAGCCAGAAACA-3′, GADPH Fwd 5′-ACGGCAAATTCAACGGCACAGTCA-3′, GAPDH Rev 5′-TGGGGGCATCGGCAGAAGG-3′, *Defa1* Fwd 5′-TCAAGAGGCTGCAAAGGAAGAGAAC-3′, Defa1 Rev 5′-TGGTCTCCATGTTCAGCGACAGC-3′, *Defa2* Fwd 5′-CCAGGCTGATCCTATCCAAA-3′, Defa2 Rev 5′-GTCCCATTCATGCGTTCTCT-3′, *Reg3g* Fwd 5′-AACAGAGGTGGATGGGAGTG-3′, Reg3g Rev 5′-GGCCTTGAATTTGCAGACAT-3′, S100A8 Fwd 5′-GGAAATCACCATGCCCTCTA-3′, S100A8 Rev 5′-ATCACCATCCGAAGGAACTC-3′, *S100a9* Fwd 5′-GTCCAGGTCCTCCATGATGT-3′, *S100a9* Rev 5′-TCAGACAAATGGTGGAAGCA-3′, *defb3* Fwd 5′-GTCTCCACCTGCAGCTTTTAG-3′, *defb3* Rev 5′-ACTGCCAATCTGACGAGTGTT-3′. The expression of target mRNA was calculated and normalized to the expression of the house keeping gene GAPDH using the 2(-ΔΔCT) method.

Relative abundance of γ-proteobacteria was analyzed by quantitative PCR using Universal SYBR Green PCR Master Mix (Bio-Rad Laboratories) and an ABI7000 (Applied Biosystems). The following oligonucleotides were used: γ-Proteobacteria 1080gF 5′-TCGTCAGCTCGTGTYGTGA-3′, g-prot g1202R 5′-CGTAAGGGCCATGATG-3′, EUB1114 Fwd 5′-CGGCAACGAGCGCAACCC-3′, EUB1221 Rev 5′-CCATTGTAGCACGTGTGTAGCC-3′. The relative abundance of γ-proteobacteria was calculated to eubacteria using the 2(-ΔΔCT) method.

### 16S rRNA Sequencing and Analysis

16S rRNA gene sequencing of ileal lumen samples were processed for DNA isolation using the QIAmp DNA Stool mini kit. The V4 region of 16S rRNA gene was PCR-amplified using barcoded primer described previously (45) and sequenced using the Illumina MiSeq Platform (2 × 250–bp paired-end reads). OTU picking was performed using UPARSE (usearch v.8.0.162) (46), and taxonomy was assigned using the uclust method with the Greengenes 13.8 database (QIIME v1.9) (47).

### Statistical Analyses

All analyses were performed with Prism 5.0 (GraphPad). Data were analyzed with a non-paired Student's *t*-test, survival data was analyzed using Log-rank Mantel-Cox test a *p* < 0.05 was considered significant.

## Ethics Statement

All animal experiments were conducted according to U.S.A. Public Health Service Policy of Humane Care and Use of Laboratory Animals. All protocols were approved by the Institutional Animal Care and Use Committee (School of Medicine, Washington University in St. Louis), based on the Guide for the care and Use of Laboratory Animals. IACUC Protocol Numbers: 20160128 and 20160220.

## Author Contributions

LC-B designed and performed experiments, analyzed data, and wrote the manuscript. VC, QW, KM, BD, and JC performed experiments and analyzed data. SG, RN, LS, and C-SH designed experiments, analyzed data, and reviewed the manuscript. MC designed experiments and wrote the manuscript.

### Conflict of Interest Statement

The authors declare that the research was conducted in the absence of any commercial or financial relationships that could be construed as a potential conflict of interest.
