# Supplementary material for: CRTAM Protects Against Intestinal Dysbiosis During Pathogenic Parasitic Infection by Enabling Th17 Maturation
Source: Front Immunol. 2019 Jul 2;10:1423. doi: 10.3389/fimmu.2019.01423 (PMC6614434; doi:10.3389/fimmu.2019.01423)
Supplement: Supplementary file 1 [file Data_Sheet_1.docx]

**Supplementary Material**

**CRTAM protects against intestinal dysbiosis during pathogenic parasitic infection by enabling Th17 maturation**

Luisa Cervantes-Barragan^1,5^, Victor S. Cortez^1^, Qiuling Wang^2^, Keely G. McDonald^3^, Jiani N. Chai^4^, Blanda Di Luccia^1^, Susan Gilfillan^1^, Chyi-Song Hsieh^4^, Rodney D. Newberry^3^, L. David Sibley^2^, and Marco Colonna^1^

^1^Department of Pathology and Immunology, ^2^ Department of Molecular Microbiology, ^3^Division of Gastroenterology, Department of Internal Medicine, ^4^Division of Rheumatology Department of Internal Medicine, Washington University School of Medicine, St. Louis, Missouri, USA.

^5^Current Address: Department of Microbiology and Immunology, Emory University. Atlanta, Georgia, USA.

Correspondence:

Luisa Cervantes-Barragan, Telephone: (404) 712-8891; FAX (404) 727 8250. email [lcervantes@emory.edu](mailto:lcervantes@emory.edu).

**
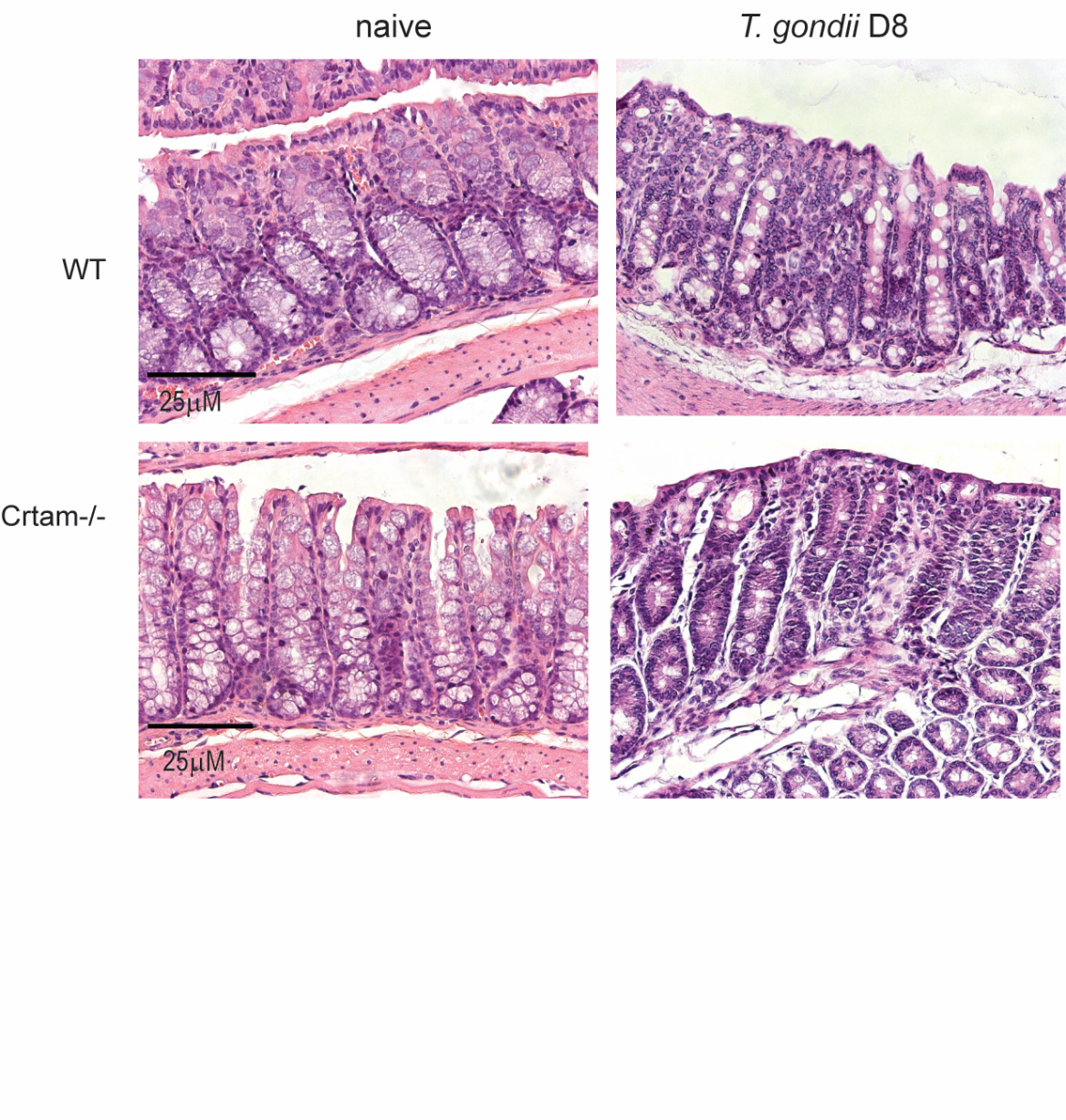
**

**Figure S1. *Crtam*^–/–^** **mice are more susceptible to ileal pathology during *T. gondii* infection.** WT and *Crtam*^–/–^ mice were infected orally with 10 cysts of *T. gondii* strain ME49, uninfected mice were used as control. Representative sections of colon stained with hematoxylin and eosin.

**
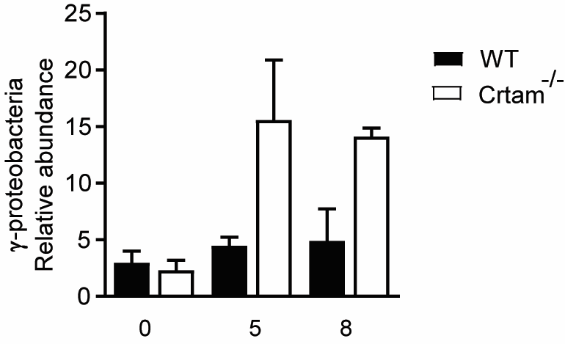
**

**Figure S2. Absence of CRTAM expression results in impaired antimicrobial peptide production.** WT and *Crtam*^–/–^ mice were infected orally with 10 cysts of *T. gondii* strain ME49. Relative abundance of γ-enterobacteria at days 0, 5, and 8 post infection.
